# The Learning Curve of Unilateral Biportal Endoscopic (UBE) Spinal Surgery by CUSUM Analysis

**DOI:** 10.3389/fsurg.2022.873691

**Published:** 2022-04-29

**Authors:** Lei Chen, Bin Zhu, Hua-zhang Zhong, Yi-guo Wang, Yi-song Sun, Qi-fei Wang, Jian-jun Liu, Da-sheng Tian, Jue-hua Jing

**Affiliations:** Department of Orthopedics, The Second Hospital of Anhui Medical University, Hefei, China

**Keywords:** unilateral biportal endoscopic spinal surgery, learning curve, lumbar disc herniation, cumulative summation, operative time

## Abstract

**Objective:**

To assess the learning curve of the unilateral biportal endoscopic (UBE) technique for the treatment of single-level lumbar disc herniation by cumulative summation (CUSUM) method analysis.

**Methods:**

A retrospective analysis was conducted to assess 97 patients' general condition, operation time, complications, and curative effect of single segmental UBE surgery performed by a spinal surgeon in his early stage of this technique. The learning curve of operation time was studied using a CUSUM method, and the cut-off point of the learning curve was obtained.

**Results:**

The operation time was 30 – 241(97.9 ± 34.7) min. The visual analog scale score of lower limb pain decreased from 5.75 ± 0.81 before the operation to 0.39 ± 0.28 at the last follow-up (*P* < 0.05). The Oswestry disability index score decreased from 66.48 ± 4.43 before the operation to 14.57 ± 3.99 at the last follow-up (*P* < 0.05). The CUSUM assessment of operation time revealed the learning curve was the highest in 24 cases. In the learning stage (1–24 cases), the operation time was 120.3 ± 43.8 min. In the skilled stage (25–97 cases), the operation time was 90.5 ± 27.8 min.

**Conclusions:**

About 24 cases of single segmental UBE operation are needed to master the UBE technique.

## Introduction

Lumbar disc herniation is a common disease that presents as low back pain, lower limb pain, numbness, weakness, or claudication, with a lifetime prevalence of 12.2–43% (1). Conservative treatment can be tried for patients with mild symptoms and without progressive decline (2). However, for patients whose conservative treatment failed, surgery may be the best option (3, 4). In recent years, unilateral biportal endoscopic (UBE) spinal surgery for the treatment of lumbar degenerative diseases and other diseases has gradually increased (5–8). It is generally believed that UBE surgery has the advantages of a wider field of vision, flexible operation, minimally invasive, and contributing to full nerve decompression and faster recovery (9).

Applicable to the most skilled spinal surgeons, there are still some difficulties and risks in the early implementation of UBE technology (10). Navigating the UBE learning curve is a concern for most surgeons who wish to use this technology. At present, there are still few studies on the learning curve of UBE technology. We adopt the cumulative summation (CUSUM) method to analyze the relationship between the number of repeated operations using UBE technology and the possibility of a successful single operation to provide a quantitative basis for determining the optimal number of repetitions in the learning process (11). In addition, the potential methods to shorten the learning curve of UBE were empirically summarized. Through all these, it may provide some references for doctors interested in performing UBE surgery.

## Methods

This retrospective study was conducted in accordance with the guidelines of the Declaration of Helsinki and was approved by the ethics committee of the Second Hospital of Anhui Medical University (No. SL-YX2018-324(F1)). Patients with single segmental lumbar disc herniation treated in the Department of Orthopedics of the second Hospital of Anhui Medical University from November 2018 to May 2020 were studied, and UBE spinal surgery was performed entirely by the same doctor. All patients signed the informed consent form according to the standard of diagnosis and treatment before operation. The inclusion criteria that were used are as follows: (1) patients with single-segment lumbar disc herniation, who have clear surgical indications, (2) American Society of Anesthesiology (ASA) levels I–III, and (3) complete follow-up data can be obtained and the follow-up period is at least 18 months. The exclusion criteria are as follows: (1) patients with extreme lateral, very middle, or bilateral disc herniation, (2) patients with other serious diseases, (3) patients with previous lumbar surgery history, (4) patients with lumbar instability, lumbar infection, or lumbar tumor, (5) patients with the multisegmental lumbar disease need to be treated, (6) a patient whose operation is performed by another doctor. According to the above inclusion and exclusion criteria, a total of 97 patients were enrolled in this study.

### Surgical Technique

The patient underwent general anesthesia and was placed in the prone position. With C-arm fluoroscopy, adjustments to the operating bed were made, so that the target intervertebral space is as perpendicular to the ground as possible. Taking the intersection of the upper and lower 1–1.5 cm of the target intervertebral space and the inner edge of the pedicle as the center, a 1–1.5 cm transverse incision was made. The left-hand incision serves as the observation channel (portal), and the right-hand incision serves as the working channel. The bilateral channels were dilated with a step-by-step dilator and the lower edge of the superior lamina and the interlaminar space can be touched by the dilator. The operator held the arthroscope in his left hand and the instrument in his right hand. Through the two channels, the camera lens and instrument will meet in the space around the interlaminar window in a continuous perfusion water environment.

**Figure 1:**
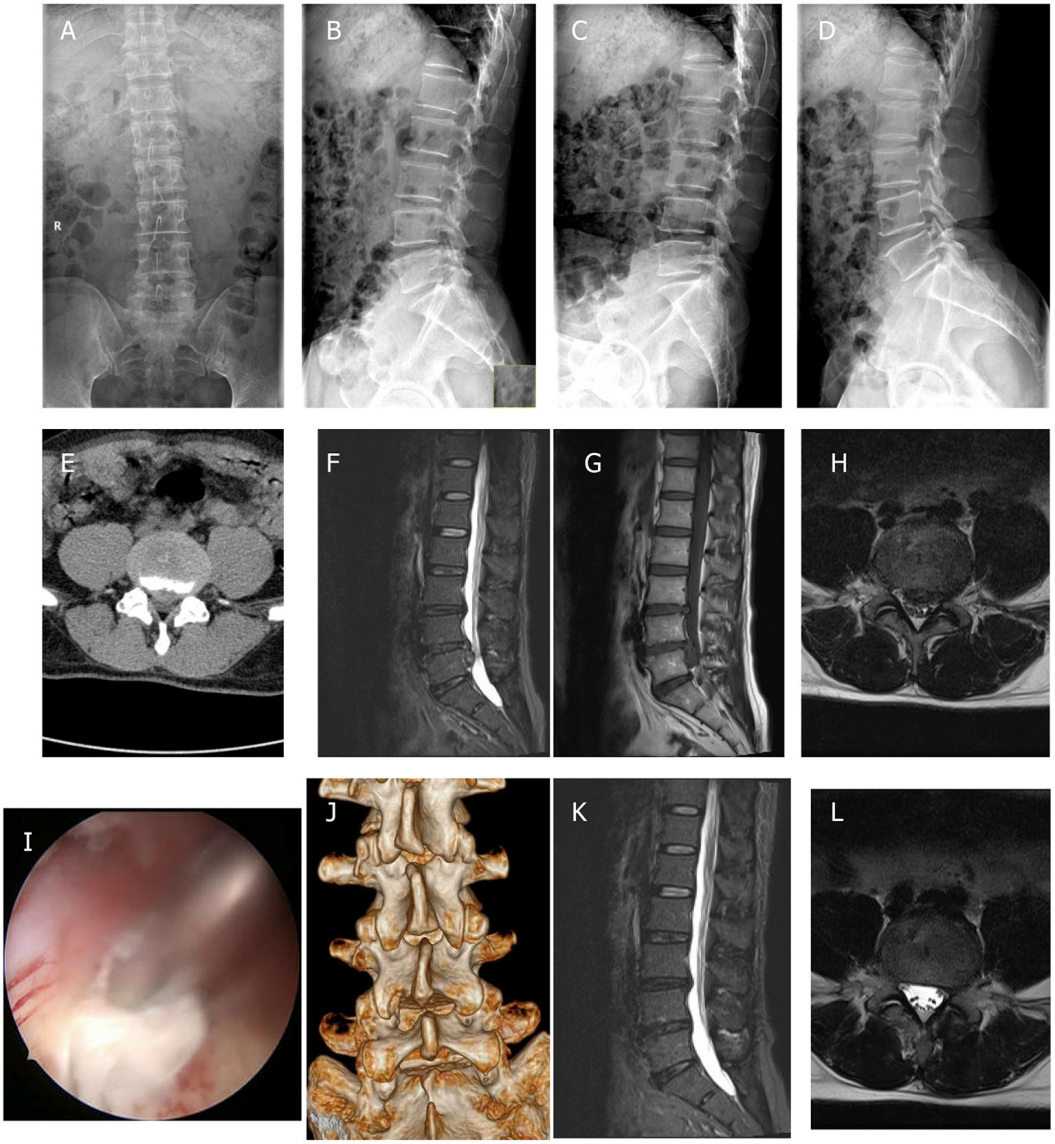
Unilateral biportal endoscopic (UBE) diskectomy was performed on a 47-year-old male patient with L4/5 lumbar disc herniation. **(A,B)** Preoperative anteroposterior and lateral plain radiographs; **(C,D)** preoperative flexion and extension radiographs; **(E)** preoperative CT scans; **(F–H)** preoperative MRI scans; **(I)** diskectomy was performed to ensure adequate decompression of the nerve tissue; **(J)** postoperative 3D-CT scans; and **(K,L)** postoperative MRI scans.

The structures such as the inferior edge of the superior lamina, the root of the spinous process, the upper edge of the inferior lamina, the inner edge of the facet joint, and interlaminar ligamentum flavum were exposed using a plasma radio-frequency knife. Tools such as the power grinding drill, osteotome, and gun rongeur were used to remove bones of, for example, the lower edge of the upper lamina, the upper edge of the lower lamina, and the medial side of the facet joint. Then the ligamentum flavum was removed. The intervertebral disc that compressed the nerve was explored and removed. After confirming that there was no nerve compression or active bleeding, the instrument was removed and the incision was closed. A representative case is shown in Figure 1.

Surgeon's experience: the surgeon, a senior orthopedic (spinal surgery subspecialty) doctor, independently completed more than 500 single-portal spinal endoscopic operations and more than 1,000 lumbar open decompression operations before starting these cases, and completed spinal minimally invasive (including UBE) related training in a number of spinal centers. The first assistant is one of two regular spinal surgeons.

### Observation Indicators

(1) General patient demographics and condition: age, sex, and underlying disease; (2) preoperative-related indexes: duration of preoperative symptoms, preoperative visual analog scale score (VAS), Oswestry disability index score (ODI), and target segment dural sac area; (3) indexes related to operation: operation time and amount of bleeding; (4) postoperative-related indicators: postoperative hospital stay, VAS score, ODI score, Macnab grade (the patient is asked to rate his level of wellbeing, generally after surgery; the patient choose one of the four: (1) excellent, (2) good, (3) fair, and (4) poor) (12), and target segment dural sac area, complications, and reoperation.

### Statistical Analysis

SPSS20.0 software was used for statistical analysis. The independent samples Student's *t*-test was used to compare the measurement data between groups, and the paired samples Student's *t*-test was used to compare the measurement data before and after the operation. The chi-square test was used to compare categorical parameters. Significance was assigned at *P* < 0.05.

The learning curve was analyzed by CUSUM analysis. The formula is as follows: $CUSUM=\overset{n}{\underset{i=1}{\sum }}\left(Xi-u\;\right)$. *Xi* indicates the actual operation time for each patient and *u* indicates the average operation time of this group of patients. The difference between the operation time of each patient in chronological order and the average operation time of the whole group was summed and the learning curve was obtained.

## Results

There were 52 men and 45 women. The age was 21.0–86.0 (51.5 ± 15.4) years old. The body mass index was 16.1–31.6 (23.9 ± 4.8) kg/m^2^. The duration of preoperative symptoms was 1–240 (24.4 ± 39.5) months. Among the 97 patients, 19 cases were complicated with hypertension, diabetes, old cerebral infarction, rheumatoid, etc. The detailed demographic data are presented in Table 1.

**Table 1:** Demographic factors of patients included in this study.

**Characteristic**	**Value**
Patients (*n*)	97
Age (years)	
Mean ± SD	51.5 ± 15.4
Range	21–86
Sex (*n*)	
Male	52
Female	45
Body mass index (kg/m^2^)	
Mean ± SD	23.9 ± 4.8
Range	16.1–31.6
Operative level (*n*)	
L3/4	9
L4/5	40
L5/S1	48
Patents with basic disease (*n*)	19
Duration of symptoms (months)	
Mean ± SD	24.4 ± 39.5
Range	1–240

All 97 patients underwent the UBE operation successfully. The follow-up time was 18–36 (22.6 ± 3.6) months. The operation time was 30–241 (97.9 ± 34.7) min. The estimated intraoperative blood loss was 10–50 (20.4 ± 5.0) ml. The postoperative hospital stay was 1–14 (4.4 ± 2.1) days. The VAS score of lower limb pain decreased from 5.75 ± 0.81 before the operation to 0.39 ± 0.28 at the last follow-up (*P* < 0.05). The ODI score decreased from 66.48 ± 4.43 before the operation to 14.57 ± 3.99 at the last follow-up (*P* < 0.05). The postoperative MacNab grade was grade 1 in 84 cases (86.6%), grade 2 in 7 cases (7.2%), grade 3 in 6 cases (6.2%), and grade 4 in 0 cases. The area of the dural sac at the narrowest part of the target segment increased from 89.34 ± 32.85 mm^2^ to 140.86 ± 39.87 mm^2^ (*P* < 0.05).

During the follow-up, 4 complications occurred. Complications of dural injury were found in 2 cases, of which 1 case was managed by wound expansion and suture in the later stage of incision eminence and exudation. A total of 2 cases of residual nerve compression of intervertebral disc herniation were cured by percutaneous transforaminal endoscopic discectomy surgery. The preoperative and postoperative characteristics of the whole group of cases are listed in Table 2.

**Table 2:** Preoperative and postoperative characteristics of the whole cohort.

**Characteristic**	**Preoperative**	**Last follow-up**	*P*-value^*^
Leg VAS	5.75 ± 0.81	0.39 ± 0.28	<0.001
ODI	66.48 ± 4.43	14.57 ±3.99	<0.001
Sac cross-sectional area	89.34 ± 32.85	140.86 ± 39.87	<0.001
Macnab criteria			
1 (Excellent)		84 (86.6%)	
2 (Good)		7 (7.2%)	
3 (Fair)		6 (6.2%)	
4 (Poor)		0 (0%)	

*Data presented as mean ± SD for numerical parameters and as n (%) for categorical parameters*.

* *Statistical analyses were performed between the preoperative and postoperative characteristics by paired samples student t-test*.

**Figure 2:**
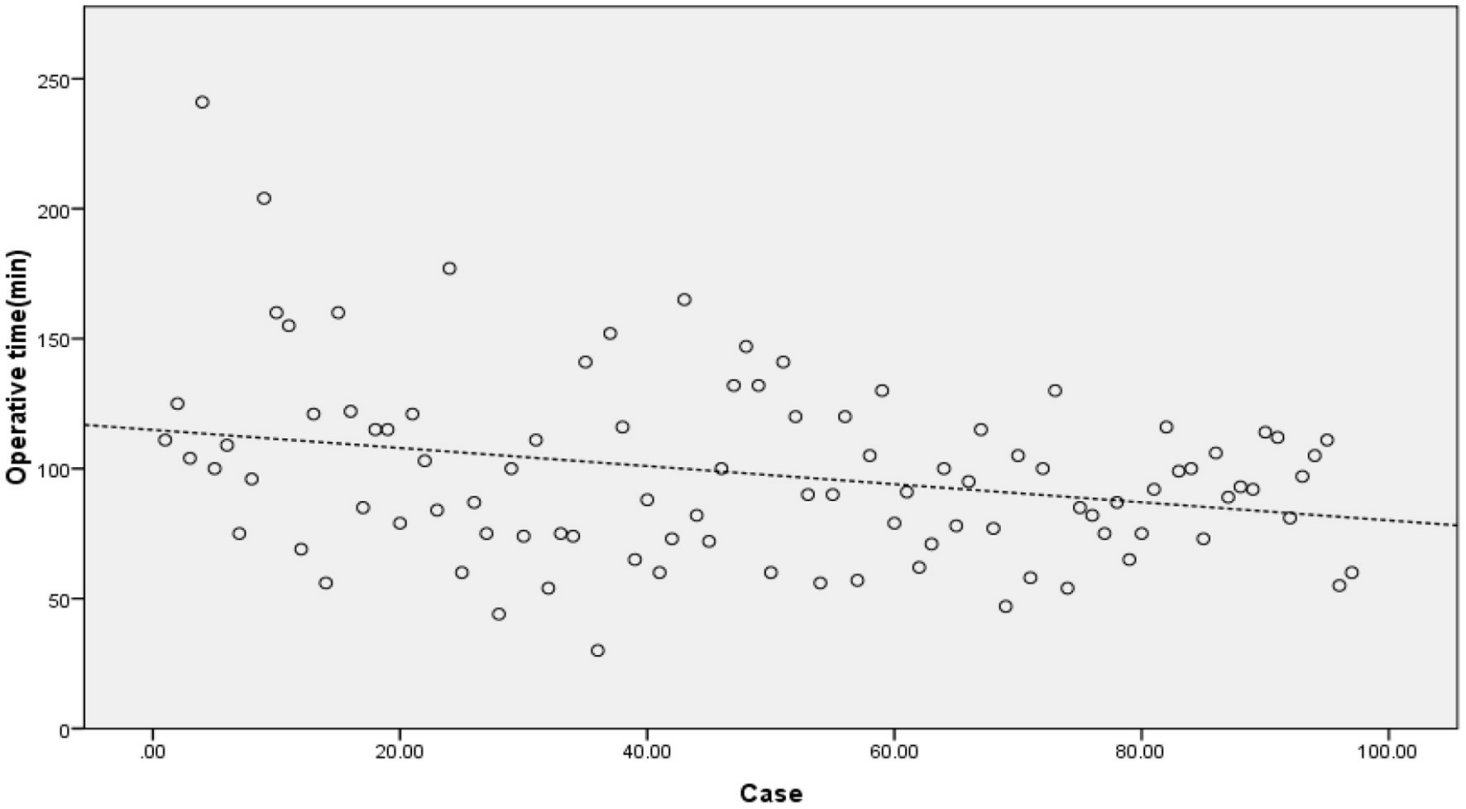
The scatter chart of the operation time showed a downward trend. The dashed line was automatically linear fitted by SPSS software.

**Figure 3:**
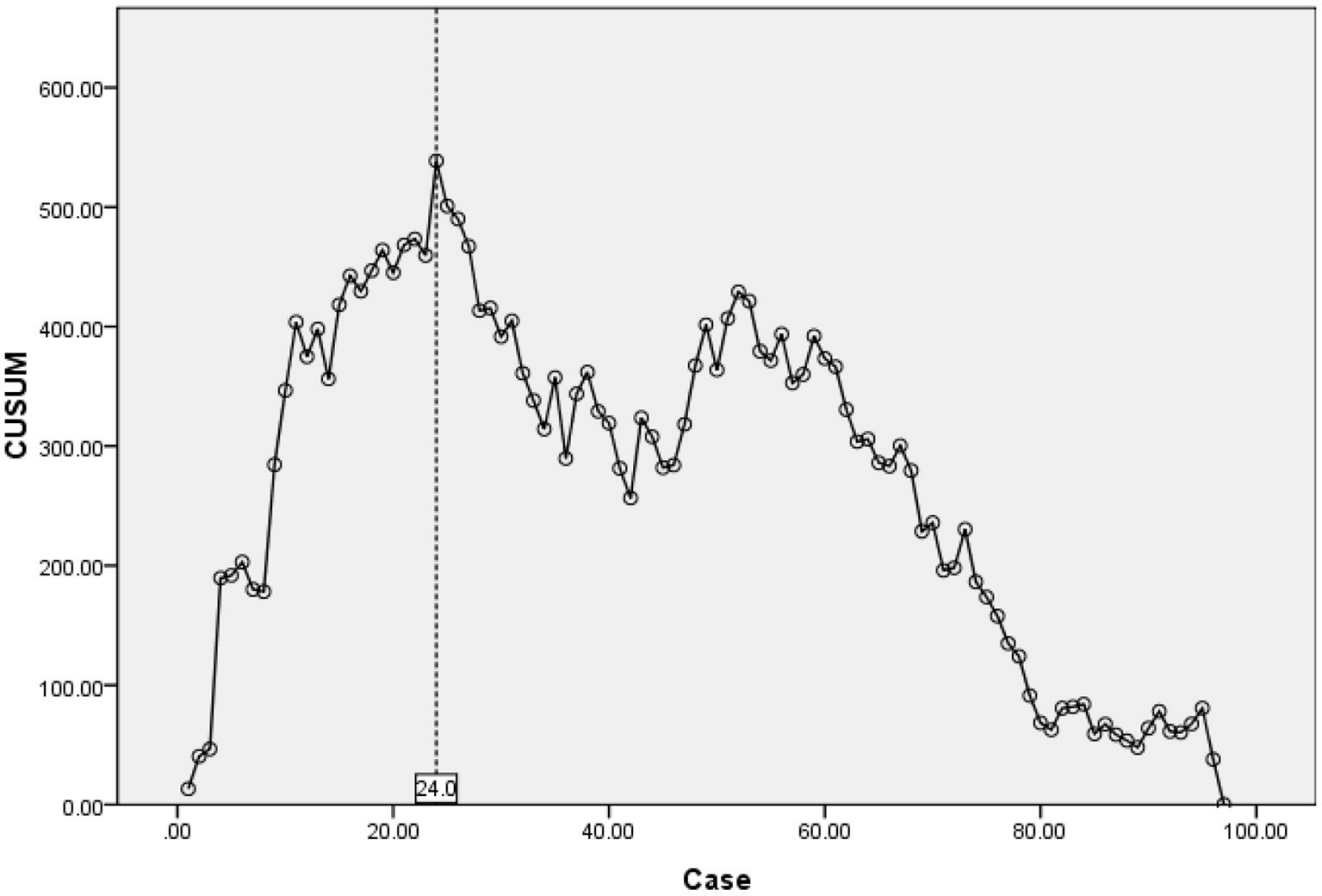
CUSUM for learning curve reached the maximum in no. 24 case, revealed competency after 24 cases.

The operation time showed a downward trend as a whole. The scatter chart of the operation time is shown in Figure 2. The CUSUM analysis curve of the learning curve is shown in Figure 3. CUSUM method showed that the curve reached the maximum in the no. 24 case, and then decreased gradually. So the cut-off point of the learning curve was selected as 24 cases. According to the cut-off point, the curve could be divided into two stages: the first stage was the learning stage in which the CUSUM value was increasing (the first 24 cases), and the latter stage was the proficiency stage in which the CUSUM value gradually decreased (after 24 cases).

Comparison of general data between the two stages: there was no significant difference between the two stages in terms of sex, age, body mass index, preoperative complications, duration of preoperative symptoms, preoperative lower limb VAS score, preoperative ODI score, preoperative dural sac area, and operative level (*P* > 0.05). The general characteristics stratified by learning period are listed in Table 3.

**Table 3:** General characteristics stratified by learning period.

**Characteristic**	**Early cases (1–24)**	**Late cases (25–97)**	*P*-value^*^
Patients (*n*)	24	73	
Age (years)	50.5 ± 15.9	51.9 ± 15.3	0.703
Sex (male to female)	10:14	42:31	0.239
Body mass index (kg/m^2^)	23.3 ± 5.2	24.1 ± 4.7	0.386
Patents with basic disease (*n*)	4 (16.7%)	15 (20.5%)	0.465
Preoperative duration of symptoms (months)	19.1 ± 31.5	26.2 ± 41.8	0.447
Preoperative leg VAS	5.55 ± 0.81	5.81 ± 0.80	0.161
Preoperative ODI	65.71 ± 4.90	66.74 ± 4.27	0.325
Preoperative sac cross-sectional area	91.80 ± 25.90	88.53 ± 34.83	0.674
Operative level (L3/4:L4/5:L5/S1)	2:11:11	7:29:37	0.102

*Data presented as mean ± SD for numerical parameters, and as n (%) for categorical parameters*.

* *Statistical analyses were performed between early and late groups*.

Comparison of the clinical effects of the two stages: the operation time, postoperative hospital stay, and the proportion of Macnabcriteria1 grade in the second stage were improved from the first stage (*P* < 0.05). There was no significant difference in the incidence of postoperative complications, VAS, ODI, and postoperative dural sac area between the two stages (*P* > 0.05). The clinical effect characteristics according to the learning period are listed in Table 4.

**Table 4:** Clinical effect characteristics stratified by learning period.

**Characteristic**	**Early cases (1–24)**	**Late cases (25–97)**	*P*-value^*^
Patients	24	73	
Operative time (min)	120.3 ± 43.8	90.5 ± 27.8	0.004
Postoperative hospital stay (days)	4.7 ± 2.4	3.9 ± 1.8	0.037
Complications (*n*)	2 (8.33%)	2 (2.74%)	0.255
Last follow-up leg VAS	0.35 ± 0.31	0.37 ± 0.27	0.783
Last follow-up ODI	11.21 ± 3.82	15.67 ± 3.40	0.104
Last follow-up sac cross-sectional area	147.84 ± 44.45	138.57 ± 38.30	0.326
Macnab criteria I(Excellent)	18 (75.0%)	66 (90.4%)	0.044

*Data presented as mean ± SD for numerical parameters and as n (%) for categorical parameters*.

* *Statistical analyses were performed between early and late groups*.

## Discussion

The ideal management strategy for lumbar disc herniation remains controversial (13). Surgical treatment is a common method of treatment, which can be more effective than conservative treatment in patients with severe lumbar spinal nerve compression (14). With the main purpose of surgery to relieve nerve compression, there are many ways of performing the operation, namely, open surgery, microscopic surgery, and percutaneous transforaminal endoscopic surgery (15). Decompression combined with internal fixation is not superior to simple decompression in many cases (16). In recent years, there have been increasing reports of UBE surgery for lumbar disc herniation and lumbar spinal stenosis (5–8). In addition, the UBE technique can be used for nerve decompression of burst fracture (17), excision of the perispinal cyst (18, 19), clearance of epidural abscess (20), treatment of epidural lipomatosis (21), treatment of foraminal stenosis (8, 22), lumbar interbody fusion (6), and revision surgery (23).

The unilateral biportal endoscopic (UBE) technique uses an independent working channel, which can achieve complete decompression under a wide visual field of the arthroscopy (9). During the making of the channel, there is no need to strip away too much soft tissue. The lens and instrument are operated directly through soft tissue channels to the target. We found in our practice that even for obese patients, no significant difficulty was increased in the surgery. The channel provides less restriction for instrument movement, and the continuous perfusion of saline during the operation is a major advantage for infection prevention. Compared with microscope technology, UBE technology has a higher success rate, shorter operation time and hospital stay (24). In our study, 86.6% of patients got MacNab grade 1. Most surgeons choose a 30-degree arthroscopic lens, which can be used to observe the lateral structure of the lens because of its wide field of view (25). UBE technique can allow visualization of the contralateral spinal canal and intervertebral foramen (5). Compared with percutaneous transforaminal endoscopic surgery, the UBE technique has less radiation exposure (26). The injury of the multifidus muscle after UBE is minimal (27). Other advantages of the UBE technique are less destruction of the facet joint, lower incidence of complications, a lesser degree of postoperative back pain, and higher satisfaction (24, 28–30). UBE technique can essentially be used as an alternative to the microscope technique (31, 32). Compared with microscopic surgery, endoscopic surgery such as UBE has been found to contribute to less pain in the early stage after the operation (33). In addition, the implementation of UBE technology does not require the purchase of special lenses and instruments as seen in the percutaneous transforaminal endoscopic technique. UBE can use general arthroscopic lenses and open spinal surgical instruments, which is more conducive to wide acceptance in most hospitals.

The unilateral biportal endoscopic (UBE) technique requires both hands to operate the lens and surgical instruments and requires sufficient coordination of both hands and stable instrument operation with a single hand. In the early stage, it is difficult for spinal surgeons who have no experience in using arthroscopic equipment to coordinate the depth and direction of the lens, move the instruments quickly and smoothly in and out of the instrument channel and quickly acquire the field of vision. If the operation is performed incorrectly, complications such as dural injury often occur in UBE surgery (10). A total of 2 cases of dural injury were found in our patients, too. These have higher requirements for the surgeon's UBE technology, the cooperation of the surgical team, and the perioperative management, which have become a big obstacle to the further popularization and development of this technology. Navigating the learning curve quickly and safely is a core issue in the clinical application of UBE. At present, there are many studies on the learning curve of transforaminal endoscopy, but few studies exist on the learning curve of UBE technology.

As a new technology for the minimally invasive spine, UBE contributes a certain learning curve, which is mainly reflected in operation time and complications. CUSUM method is a quantitative analysis method for analyzing the learning curve of surgical techniques (11). Many other studies on the learning curve of surgical techniques are mostly based on the method of grouping all cases in order, which is subjective. And the cut-off point of the learning curve is often an integer multiple of the number of grouped cases, so the results are inaccurate. In our study, the CUSUM method is selected for the analysis. To obtain the operation time of each patient, the relationship between the operation time of each patient and the average value of the group is calculated, and the approximate parabola curve is obtained. At the highest point of the parabola curve, the learning curve is divided into two stages. According to the formula, the operation time of most cases before the highest point of the parabola is longer than the average operation time, and the operation time of most cases after the highest point is < the average operation time. There is no need for artificial subjective grouping in the study of the CUSUM method, which is more objective and accurate than the grouping method (34–36). According to the highest point of the CUSUM curve (Figure 3), there were 1–24 cases in the early stage of this study and 25–97 cases in the later stage. This graph reveals that the initial curve is very steep, but it does not take too many cases to reach the highest point. With the increase in the number of cases (after 24 cases), the CUSUM curve of operation time showed a downward trend to be stable in the later stage. Evidence of the gradual decrease of operation time can also be seen in the scatter chart of operation time (Figure 2). This shows that the difficulties encountered at the beginning of UBE technology, such as long operation time, are short-lived. After a period of learning and acclimation, the surgeons become more familiar with the surgical equipment and surgical procedures. Meanwhile, with the gradual optimization of the operating room procedures and the cooperation of other personnel, the learning curve gradually becomes more stable.

Through the comparison of the data of the two stages, there is no statistical difference in the general condition and preoperative index of the patients. But the operation time, postoperative hospital stay, and the proportion of Macnabcriteria1 grade in the second stage are all improved from those in the first stage, and the difference is statistically significant. This may be due to multiple reasons: the technique of the surgeon improves; the cooperation of fixed assistants gains understanding; anesthesia, nursing, and other surgical team cooperation are gradually optimized, and perioperative management is optimized. Although the operation time shortened with the learning stage, there was no significant difference in the incidence of postoperative complications, last follow-up VAS, ODI, and the area of the dural sac after operation between the two stages. This indicated that in our earliest cases, although it takes a longer time to operate, it still ensures a clinical effect and safety that is essentially the same as that in the mature stage. Looking at the CUSUM curve, it shows that in about 42–52 cases, the curve increased slightly again. This occurrence may be related to the increasing challenge of more difficult and complex cases after the surgical technique becomes proficient. Usually, as the technique is mastered, surgeons will unconsciously extend the application of the technique to more difficult cases that they may be reluctant to choose at an early stage (11). As we can see in our cases, the proportion of patients with the basic disease and the duration of preoperative symptoms in the second stage cases were higher than those in the first stage, although there was no significant statistical difference (Table 3).

What is the difference between the learning curve of the UBE technique and other invasive techniques such as percutaneous transforaminal endoscopic surgery? With regard to the learning curve of percutaneous transforaminal endoscopic surgery, the cut-off point reported in the early literature was about 40–70 cases (37, 38), while the cut-off point reported later in the new literature was about 20 cases (39, 40). However, like the early explorers of UBE technology in China, it only takes about 24 cases to master this technique skillfully, and its learning curve is shorter than that of transforaminal endoscopic surgery reported in the early literature. The shortening of the learning curve means that the operation time, hospital stay, operation costs, and complications can be reduced in a short time, which is more beneficial to patients and more likely to be recognized by surgeons. UBE technology provides the advantages of minimally invasive percutaneous transforaminal endoscopic surgery and flexible operation of open surgery, so it is currently being widely promoted in China.

What factors can optimize the learning curve? According to our experience, surgeons need rich experience in spinal surgery before carrying out this technique, and it is better to have experience in single-portal spinal endoscopy such as percutaneous transforaminal endoscopic surgery and double-portal endoscopic surgery such as arthroscopy surgery. At the same time, the surgeon must be trained in UBE technology. Our department has held UBE training using the plastic model and the fresh specimens of piglet spine many times, which is helpful for the surgeons to successfully overcome the steep learning curve of UBE technology. In the early stage, one should try to select the cases with typical, unilateral symptoms, clear surgical indications, less degeneration, less operative area complexity, and then gradually carry out the more difficult cases after gaining skill. Some special instruments needed in UBE, such as arthroscopy, plasma-mediated ablation probes, radio-frequency probes, and grinding drill, must be well prepared. Our general experience is for a right-handed surgeon to place arthroscope, water perfusion equipment, and other observation equipment on the left hand, while radio-frequency probe, grinding drill, and other energy power equipment on the right hand to avoid entanglement of the devices. Maintaining a clear field of vision requires the anesthesia team to provide an adequate degree of anesthesia, maintain normal blood pressure, and good muscle relaxation. This requires communication and coordination with the anesthesia team. During the operation, it is necessary to maintain the appropriate water pressure of the operating cavity. And one must pay attention to the appropriate perfusion pressure and the placement of the casing, and keep the effluent unobstructed at all times (41).

## Conclusions

As a new minimally invasive endoscopic technique for the spine, UBE surgery requires coordination of both hands and one-handed operation of instruments. The learning curve is steep, but a few cases (about 24 cases) are required to overcome the learning curve. If the learning curve can be navigated smoothly, this technology can provide the advantages of less surgical trauma, flexible and efficient operation under the endoscope, and rapid recovery after the operation. In this study, CUSUM analysis was used to analyze the learning curve of a single segmental UBE in the operation of lumbar disc herniation performed by the same surgeon in the early stage. The results show that after experiencing the learning curve of 24 cases, the surgeon can reach a more skilled and stable level of operation, and can significantly reduce the operation time and improve satisfaction. In summary, the steep learning curve in the early stage can be mitigated by strengthening and training before performing this operation and selecting less complex cases in the early stage.

This study does have limitations. This study analyzes cases performed by a single surgeon that already has rich experience in open and endoscopic spinal surgery before this procedure, providing a shorter learning curve, while for young surgeons with less experience, the learning curve of UBE may be longer. However, as a very early explorer of UBE in China, the surgeon can learn from less UBE experience, and the initial development is slow progress. Later operators may have more experience to learn from and may need fewer cases to overcome the learning curve. Another limitation is that only single segment lumbar disc herniation cases are selected, multisegment and other diseases were excluded. Although the use of UBE technology for other diseases may have an impact on the cut-off point of the learning curve, the vast majority of early UBE techniques are used to treat single-segment lumbar disc herniation. Therefore, this aspect should contribute little impact on the UBE learning curve.

## Data Availability Statement

The raw data supporting the conclusions of this article will be made available by the authors, without undue reservation.

## Ethics Statement

The studies involving human participants were reviewed and approved by Scientific Ethics Committee of the Second Affiliated Hospital of Anhui Medical University. The patients/participants provided their written informed consent to participate in this study.

## Author Contributions

LC: conceptualization, validation, data curation, and writing—original draft. BZ: investigation. H-zZ: software. Y-gW: methodology. Y-sS: formal analysis. Q-fW: investigation. J-jL: visualization. D-sT: methodology and formal analysis. J-hJ: conceptualization and supervision. All authors contributed to the article and approved the submitted version.

## Conflict of Interest

The authors declare that the research was conducted in the absence of any commercial or financial relationships that could be construed as a potential conflict of interest.

## Publisher's Note

All claims expressed in this article are solely those of the authors and do not necessarily represent those of their affiliated organizations, or those of the publisher, the editors and the reviewers. Any product that may be evaluated in this article, or claim that may be made by its manufacturer, is not guaranteed or endorsed by the publisher.

## Abbreviations

UBE: Unilateral biportal endoscopy
CUSUM: Cumulative summation
VAS: Visual analog scale
ODI: Oswestry disability index.
